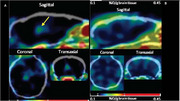# Visualization of accumulated, physiologically generated Dutch‐type Aβ oligomers associated with impaired learning and abnormal presynaptic physiology but no detectable neuroinflammation

**DOI:** 10.1002/alz70862_110303

**Published:** 2025-12-23

**Authors:** Sam E Gandy, Emilie L Castranio, Merina Varghese, Elentina K Argyrousi, Kuldeep Tripathi, Linda Söderberg, Erin Bresnahan, David Lerner, Francesca Garretti, Hong Zhang, Jonathan Van de Loo, Bram Teunissen, Ronan Talty, Efrat Levy, Minghui Wang, Bin Zhang, Lars Lannfelt, Charles G Glabe, William D Lubell, Brigitte Guerin, Shai Rahimipour, Dara Dickstein, Ottavio Arancio, Michelle E. Ehrlich

**Affiliations:** ^1^ Icahn School of Medicine at Mount Sinai, New York, NY USA; ^2^ James J. Peters Veterans Affairs Medical Center, Bronx, NY USA; ^3^ Columbia University, New York, NY USA; ^4^ Bar‐Ilan University, Raman Gat Israel; ^5^ BioArctic AB, Stockholm Sweden; ^6^ Icahn School of Medicine at Mount Sinai, NEW YORK, NY USA; ^7^ Icahn School of Medicine Mount Sinai, New york, NY USA; ^8^ Yale University, New Haven, CT USA; ^9^ Nathan S. Kline Institute for Psychiatric Research, Orangeburg, NY USA; ^10^ New York University Grossman School of Medicine, New York, NY USA; ^11^ Department of Genetics and Genomic Sciences, Icahn School of Medicine at Mount Sinai, New York, NY USA; ^12^ Icahn Genomics Institute, Icahn School of Medicine at Mount Sinai, New York, NY USA; ^13^ Ronald M. Loeb Center for Alzheimer's Disease, New York, NY USA; ^14^ Mount Sinai Center for Transformative Disease Modeling, New York, NY USA; ^15^ Icahn Institute for Data Science and Genomic Technology, New York, NY USA; ^16^ Mount Sinai Center for Transformative Disease Modeling, Icahn School of Medicine at Mount Sinai, New York, NY USA; ^17^ BioArctic, Stockholm Sweden; ^18^ University of California Irvine, Irvine, CA USA; ^19^ University of Montreal, Montreal, QC Canada; ^20^ University of Sherbrooke, Sherbrooke, QC Canada; ^21^ Bar Ilan University, Raman Gat Israel; ^22^ Uniformed Services University of Health Science, Bethesda, MD USA; ^23^ The Taub Institute for Research on Alzheimer's Disease and the Aging Brain ‐ Columbia University, New York, NY USA

## Abstract

**Background:**

Studies of Alzheimer's disease have demonstrated that cognitive decline fails to correlate with fibrillar Aβ burden.

**Method:**

We created a transgenic mouse overexpressing Dutch mutant hAPP (APPE693Q) driven by a pan‐neuronal Thy1 promoter.

**Result:**

Accumulation of oligomeric Aβ (oAβ) and alpha‐CTFs (but not Aβ fibrils) was observed in the brains of Dutch mice which develop impaired learning behavior proportional to brain oAβ levels. Male & female Dutch mice & WT controls were compared using learning behavior, ICC, transmission electron microscopy (TEM), electrophysiology, epitomic assays & single cell RNA sequencing. Brain levels of nonfibrillar oAβ in Dutch mice increased during aging as revealed by A11 ICC and FITC‐cyclic peptide (FITC‐CP) fluorescence microscopy. Electrophysiology of hippocampal synapses in Dutch and WT mice at ∼7 & ∼11 months revealed no change in basal excitatory transmission consistent with normal density & morphology of synapses in hippocampal CA1. One exception was increased postsynaptic density area in Dutch mice. Functional characterization of presynaptic termini showed abnormal post‐tetanic potentiation, synaptic fatigue & vesicle replenishment in Dutch mice. Single cell RNA‐seq to elucidate cell‐type specific transcriptional responses to oAβ revealed altered transcriptional profiles in multiple cell types. Unexpectedly, no obvious transcriptomic differences existed between Dutch vs WT microglia. Excitatory neurons showed the most altered profile which was associated with 'protein translation' & 'oxidative phosphorylation'. Mitochondrial complex I activity was reduced in 12‐ but not 7‐mo‐old Dutch vs WT mice. Ultrastructural analysis of excitatory presynaptic mitochondria revealed fewer mitochondria in Dutch mouse presynaptic termini. Nonfibrillar oAβ deposits were revealed by co‐localization of A11 immunoreactivity with FITC‐CP microscopy. Oligomer‐detecting cyclic azaglycine PET tracer Lys(64Cu/NOTA)]‐CP revealed robust PET signal from thalami and cerebral cortices of presymptomatic 5xFAD mice (10.1073/pnas.2210766119). Analysis using TEM and CP‐gold nanoparticle labeling revealed that oAβ was concentrated around mitochondria & ER in Dutch mice.

**Conclusion:**

Dutch oAβ accumulation associates with aging‐related defects in learning behavior, presynaptic function & mitochondrial structure & function. Brain PET imaging with Lys(64Cu /NOTA)]‐CP may enable development of an assay for monitoring oAβ levels & distribution for diagnosing living human subjects & patients.